# Assessment of vector/host contact: comparison of animal-baited traps and UV-light/suction trap for collecting *Culicoides *biting midges (Diptera: Ceratopogonidae), vectors of Orbiviruses

**DOI:** 10.1186/1756-3305-4-119

**Published:** 2011-06-27

**Authors:** Elvina Viennet, Claire Garros, Renaud Lancelot, Xavier Allène, Laëtitia Gardès, Ignace Rakotoarivony, Didier Crochet, Jean-Claude Delécolle, Catherine Moulia, Thierry Baldet, Thomas Balenghien

**Affiliations:** 1 CIRAD, UMR Contrôle des maladies, F-34398 Montpellier, France; 2 INRA, UE1277 PFIE, Plate Forme d'Infectiologie Expérimentale, F-37380 Nouzilly, France; 3 UdS, IPPTS, Faculté de Médecine, F-67000 Strasbourg, France; 4 Université de Montpellier 2, ISEM équipe « Interactions », F-34000 Montpellier, France

## Abstract

**Background:**

The emergence and massive spread of bluetongue in Western Europe during 2006-2008 had disastrous consequences for sheep and cattle production and confirmed the ability of Palaearctic *Culicoides *(Diptera: Ceratopogonidae) to transmit the virus. Some aspects of *Culicoides *ecology, especially host-seeking and feeding behaviors, remain insufficiently described due to the difficulty of collecting them directly on a bait animal, the most reliable method to evaluate biting rates.

Our aim was to compare typical animal-baited traps (drop trap and direct aspiration) to both a new sticky cover trap and a UV-light/suction trap (the most commonly used method to collect *Culicoides*).

**Methods/results:**

Collections were made from 1.45 hours before sunset to 1.45 hours after sunset in June/July 2009 at an experimental sheep farm (INRA, Nouzilly, Western France), with 3 replicates of a 4 sites × 4 traps randomized Latin square using one sheep per site. Collected *Culicoides *individuals were sorted morphologically to species, sex and physiological stages for females. Sibling species were identified using a molecular assay. A total of 534 *Culicoides *belonging to 17 species was collected. Abundance was maximal in the drop trap (232 females and 4 males from 10 species) whereas the diversity was the highest in the UV-light/suction trap (136 females and 5 males from 15 species). Significant between-trap differences abundance and parity rates were observed.

**Conclusions:**

Only the direct aspiration collected exclusively host-seeking females, despite a concern that human manipulation may influence estimation of the biting rate. The sticky cover trap assessed accurately the biting rate of abundant species even if it might act as an interception trap. The drop trap collected the highest abundance of *Culicoides *and may have caught individuals not attracted by sheep but by its structure. Finally, abundances obtained using the UV-light/suction trap did not estimate accurately *Culicoides *biting rate.

## Background

*Culicoides *biting midges (Diptera: Ceratopogonidae) are among the smallest hematophagous insects and a pest to livestock and humans [1]. They also can transmit several important *Orbivirus *(Reoviridae) such as African horse sickness virus to equids or bluetongue virus (BTV) to ruminants [1]. Bluetongue was considered an exotic disease in Europe until the spread of multiple BTV strains throughout the Mediterranean Basin from 1998 to the present day, mainly in association with the presence of *Culicoides imicola *Kieffer, the main Afro-tropical vector species [2]. During 2006, a BTV8 epizootic occurred in five member states of north-western Europe in the absence of *C. imicola *confirming that some autochthonous Palaearctic *Culicoides *species are able to transmit BTV [3]. However, the virus quickly spread to other countries in the following years infecting a surprising number of farms through Europe (for instance about 27,000 BTV8 and 5,000 BTV1 outbreaks in the French mainland in 2008) leading to disastrous consequences for the livestock industry with huge economic losses [4].

Many aspects of *Culicoides *ecology remain unknown especially for species suspected to be involved in BTV8 transmission in Europe [1,5]. Their host-seeking and feeding behaviors are poorly described partially because of the difficulty in collecting these small insects directly on animals. Direct collection from animals is considered the most reliable method to study the vector/host ratio [6], an essential parameter to model vectorial capacity and virus transmission [7]. Only a few collections of Palaearctic *Culicoides *have been carried out directly on hosts [8-13]. Direct aspiration and drop trap, the most common host-baited collection, have been compared to artificially baited traps especially the ultraviolet (UV) light/suction trap [12,13], which is the most widespread method to collect *Culicoides*. UV-light/suction traps seemed to underestimate biting rates of *Culicoides chiopterus *(Meigen) [12], *Culicoides obsoletus *(Meigen) and *Culicoides parroti *Kieffer [13] and to overestimate the biting rate of *C. imicola *[13] on sheep. However host-baited traps have never been compared with each other, or simultaneously to a light trap.

The aim of this study was to improve our ability to accurately describe the *Culicoides *ecology by identifying the best trapping assessment of the vector biodiversity and the biting rate in north-western Europe. We compared standard animal-baited traps (drop trap and direct aspiration), to a novel trapping system which utilized sticky panels and to a UV-light/suction trap commonly used in *Culicoides *surveillance.

## Materials and methods

Our strategy was to use a randomized Latin square design to compare the assessment of the biting rate by each animal-baited trap and to identify potential bias when UV-light/suction trapping might be used to estimate the biting rate.

### Description of collection methods

**Figure 1:**
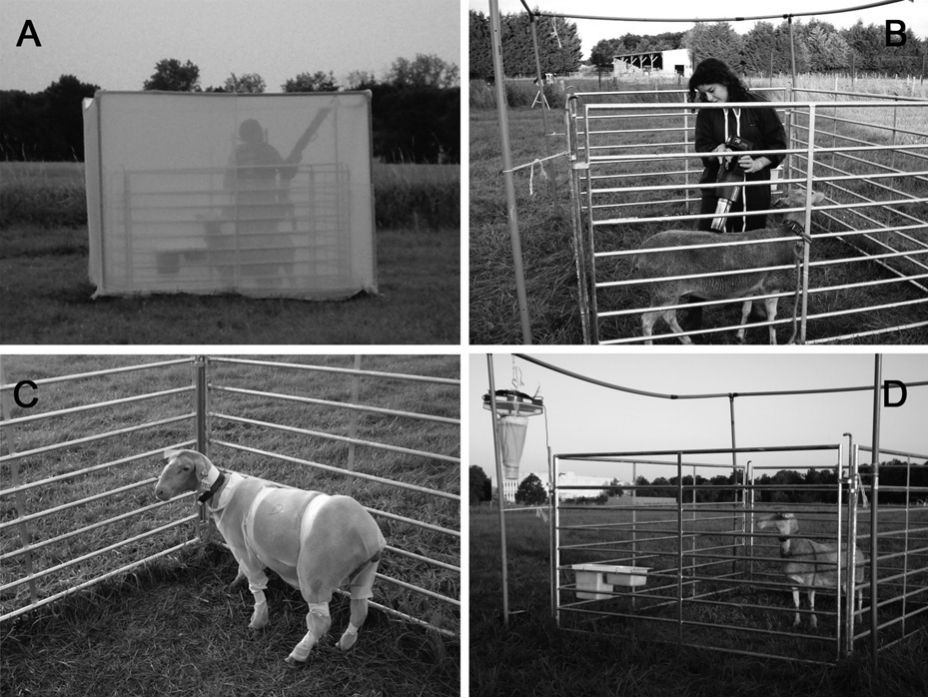
**Four collection methods compared during 12 days using a 4 × 4 Latin square design: (A) drop trap, (B) direct aspiration, (C) sticky cover trap and (D) UV-light/suction trap (OVI)**.

Four collection methods were compared during this trial (Figure 1).

The drop trap (DT) consists in a rectangular cage in shape (2.5 m wide × 3 m long and 2 m high) recovered with white polyester netting (< 0.25 mm^2 ^mesh size) [12]. Initially, a single sheep is exposed for 10 min with the netted sides of the cage raised and the observer remaining at a distance of approximately 50 m from the trap. Thereafter, the observer returns to lower the net, making sure that no insect can enter or escape, and left the site for another 10 min. Then, the observer entered the netted cage and vacuums the 4 sides plus the roof for about 10 min using a modified CDC Backpack aspirator (model 1412, John W. Hock company, http://www.johnwhock.com) with fine mesh cups (adapted to biting midges with < 0.25 mm^2 ^mesh size) to collect any *Culicoides *present. On completion of this period, the drop net is raised for an additional 10 min exposure period.

The second type of animal-baited trap assessed consisted in direct aspiration (DA) on an animal. A single sheep tethered to a cage was exposed for 10 min - the observer remaining at a distance of approximately 50 m from the animal. Then the observer vacuumed the animal using an aspirator (Heavy Duty Hand-Held Vac/Aspirator #2820GA by BioQuip Products Inc., http://www.bioquip.com) for 10 min (5 min to the upper part, from the nostrils to the tail limited by the line breast-quarter, and 5 min to the lower part, down to the line breast-quarter; the part first vacuumed was alternatively the upper and the lower part). Both sides of the sheep were completely vacuumed. Then, an additional 10 min exposure period started.

A sticky cover trap (SCT) was also developed as a new host-baited collection method. Different adhesive products were tested on a white mosquito net of fiberglass (1 mm^2 ^mesh size) for their ability to capture insects and to keep them in good condition for identification. According to the number and size of collected insects and the facility of removing and identifying them, petroleum jelly (Transgel 110^® ^AIGLON S.A.) was preferred to glue, or oil as an adhesive. This product was still sticky after several hours of exposure. Thereafter, to the panels were fitted directly onto a sheep. A mosquito netting cover was subdivided into several body parts (back, belly/flank, head, and legs) to identify the landing zones. The cover was held down on the sheep by tape and coated by a thin layer of petroleum jelly. The sheep was then allowed to move freely within the pen during each evening experiment without any human interference. At the end of the exposure period, the sticky cover was cut off carefully according to the defined body parts. *Culicoides *were then carefully removed using a paintbrush dipped in clean engine oil to dilute petroleum jelly.

We compared these host-baited traps to a UV-light/suction trap (OVI) manufactured by the Onderstepoort veterinary institute (South Africa) [14]. This trap is equipped with an 8-W UV light tube and a downdraft suction motor ended by a plastic beaker containing a drop of soap in water. It was operated with a 12-volt car battery and placed at 1.5 m height from the ground on the cage where a single sheep was present.

### Study site and procedure of trap comparison

**Figure 2:**
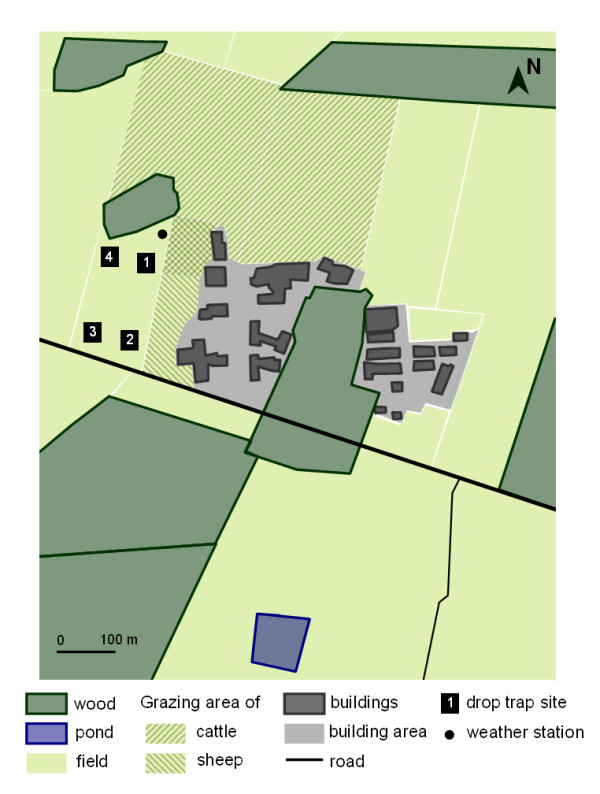
**Sketch map of the study site (Inra) at Nouzilly (western France)**.

Trap comparisons were conducted over 12 days from the 11^th ^June to 13^th ^July 2009 (a seasonally favorable period for *Culicoides *diversity and abundance in western France) on an experimental farm (Institut National de la Recherche Agronomique, INRA, UE1277 PFIE) breeding sheep and dairy cattle at Nouzilly (47°33'01''N; 00°47'52''E; western France). Four sites were designated in the field surrounded by two grazing sheep herds (10 ewes and 20 rams close to the site 2), by grazing cattle herds (10 to 20 Holstein-Friesians heifers) and by dairy/sheep holding (< 150 m) (Figure 2). Designated sampling sites were separated by 50 m to minimize interference between traps.

Traps were compared using a 4 × 4 Latin square design, replicated 3 times. Each trap was randomly assigned to a site for the first collection evening. Then, random assignation was repeated the next three evenings with the condition that traps should be assigned to a different site each collection. Assignation procedure was repeated independently for each replicate. All collections were started and stopped 1h45 before and after the sunset (3.5 hours of collection around sunset).

Six south-Prealpes female sheep were separated into two groups. They were about 6 years old, 50-kg live weight and shorn three weeks before the experimentation began. One group was assigned to the sticky cover trap to avoid that residual petroleum jelly on sheep, which could alter results when used in another treatment. In this group, one animal was randomly chosen for each collection day and was cleaned up with a shedding blade after each experiment. In the other group, all animals were randomly assigned to a given treatment to avoid potential individual sheep effects. Each protocol step was conducted with respect to the standard ethical rules (staff was qualified for animal experimentation, premises are licensed for experiment, protocol procedure does not cause any pain (no injection, no biological sample, no surgery)).

Meteorological conditions (air temperature, wind speed and direction, relative humidity and rainfall) were recorded every fifteen minutes using a weather station Vantage Pro 2 (Davis Instruments France, http://www.davis-meteo.com) situated close to the traps (Figure 2). To prevent the drop net from tearing away and to protect the animals, experiments were stopped or cancelled when wind speed was higher than 8.5 m/s or when heavy rain disrupted operation of the drop trap. Monthly meteorological data recorded by national weather station (Météo France) at Parcay-Meslay (47°26'36''N; 00°43'36''E; 13 km from the study site) in 2009 and from 1971 to 2000 were compared to illustrate meteorological conditions during the year of collections.

### *Culicoides *identification

After captures, collection cups from drop trap and direct aspiration were stored at -20 °C overnight to kill insects. *Culicoides *collected from sticky cover trap were stored in engine oil. The insects collected with the UV-light/suction trap were stored in 70% ethanol. All *Culicoides *were morphologically identified under a stereomicroscope (Stemi 2000C ZEISS) to species level based on identification key for the Palaearctic region [15] and sorted by sex. Females were classified as nulliparous, parous [16], freshly blood-fed and gravid. When morphological identification with stereomicroscope was not possible, individuals were dissected and identified using microscopic slide preparations (ZEISS imager A.1 fluorescence microscope).

All individuals identified as belonging to the Obsoletus Group (*C. chiopterus, Culicoides dewulfi *Goetghebuer, *C. obsoletus *and *Culicoides scoticus *Downes & Kettle) were molecularly confirmed and identified following the assay developed by Nolan *et al*. [17] including primers for these four species. Genomic DNA was extracted from individual midges using Chelex resin (50 μL/*Culicoides*) [18]. Primers and PCR amplifications conditions were as described by Nolan *et al*. [17]. Different preliminary tests were made before the start of collections to check the efficiency of the molecular assay on *Culicoides *collected through petroleum jelly and engine oil. Briefly, DNA extraction and PCR amplifications were carried out on two individuals kept in (i) petroleum jelly, (ii) engine oil, with and without a cleaning step before DNA extraction. Finally, prior to DNA extraction with Chelex resin, these *Culicoides *were individually cleaned on absorbent paper, followed by soapy water, rinsed twice in purified water, and dried on paper towel. We identified molecularly the origin of blood-meals of 45 engorged females randomly sampled in drop trap and direct aspiration collections at different collection dates. The aim was to confirm that they had fed on the bait animals. Primers and PCR conditions were as described by Garros *et al*.[19].

### Statistical analysis

We compared the number of *Culicoides *females and their parity rate (proportion of parous females per collection) between traps. Exposure periods differed between traps (3 × 10 min for DA, 2 × 10 min for DT, 1 × 60 min for OVI and SCT during one hour). We assumed that *Culicoides *attracted to bait remained in the vicinity of the drop trap outside the exposure period and that some *Culicoides *remained attracted by the bait during the direct aspiration period. Thus, we did not correct the data and compared the biting rate assessed by operating each method during a given period (here 3.5 hours). For plotting, abundance data were log_e _(n + 1) transformed to limit the overwriting of some points by high values. For analysis, we used untransformed abundance data.

Data were modeled using a Poisson mixed model for abundance data of dominant species, and a Binomial mixed model for parity rates of all species [20]. Capture date and site were the crossed random effects and trap was the fixed effect. However, exploratory analysis revealed an excess of zeros with respect to a Poisson distribution in abundance data for minor species. For these species the counts were analyzed using the so-called Hurdle model to model the counts [21,22]. The Hurdle model has two components: (i) a truncated count component (Poisson regression model) was used for counts > 0, and (ii) a hurdle component modeled zero vs. larger counts (binomial regression model). Date and site effects were left in the residual variance.

Predicted values were plotted against observed counts to assess model goodness of fit. Cook's distance was used to detect influential observations [23]. For comparisons of trap effects, Wald tests were used together with Holm's *p*-value adjustment for multiple comparisons (α = 0.05). On some occasions, when the fitted probabilities are close to zero, the Wald test may give unreliable results [24]. It was the case for species of low abundance. To overcome this problem, we used a graphical procedure [25] ensuring that non- overlapping confidence intervals indicated significant statistical differences. For these particular situations, which corresponded to cases of excess of zero, we simply gave the lowest p-value for which a difference in abundance between trap types was observed (if this p-value remained compatible with an acceptable primary risk, *i.e*. α close to 0.1).

Using direct aspiration and the sticky cover trap, we collected *Culicoides *by body region: upper and lower parts for direct aspiration and back, head, belly and legs for sticky cover trap. Upper part *versus *lower part in the direct aspiration and back/head *versus *belly/legs in the sticky cover trap were compared for the five most abundant species using the chi-squared test (α = 0.05). For these species, we explored the variability of their host-seeking activity around sunset as regular collections were made using direct aspiration (10 min by each 20 min period) and drop trap (10 min by each 30 min period).

All data analyses were performed using the R statistical package [26].

## Results

### Climatic data

Climate in the study site was oceanic, with mean annual temperature of 11.4°C, thermal amplitude of 14.9°C and annual rainfall of 694 mm (Météo France data, 1971-2000). The year 2009 was a standard year in terms of meteorology except a slight water deficit in spring (195 mm between February and May 2009 *versus *234 mm for the reference period) and a dry month of August (3 mm *versus *40 mm). Due to technical problems, complete meteorological data sets were available only for 9 of 12 sunset collection periods. During these collection days, wind was mainly oriented to northwest and southeast, with maximum speed of 4.5 m/s and no rainfall was recorded. Collections were carried out with temperatures between 12.5 and 29.5°C and humidity between 45 and 95%.

### Collection data

During the 12 collection carried out around sunset, a total of 534 *Culicoides *(525 females and 9 males) belonging to 17 species were collected (Table 1). It was not possible to morphologically identify 2 damaged specimens which were recorded as *Culicoides *sp. Molecular assay confirmed morphological identification and separated sibling species for 88/90 individual from the Obsoletus Group (i.e. *C. obsoletus, C. scoticus, C. dewulfi *and *C. chiopterus*). It was not possible to identify 2 individuals from the Obsoletus Complex (i.e. *C. obsoletus *and *C. scoticus*).

**Table 1:** Numbers of *Culicoides *collected over 12 nights using four trapping methods

Species^1^	**Total No**.		Rank species	No. *Culicoides *collected with															
				Drop trap^2^				Direct aspiration			Sticky cover trap				UV-light/suction trap				
	F	M		No. F	Parity	No. E	No. M	No. F	Parity	No. E	No. F	Parity	No. E	No. G	No. F	Parity	No. E	No. G	No. M
*C. brunnicans*	313	6	1	153	0.48	49	4	36	0.69	1	69	0.39		7	55	0.67			2

*C. obsoletus*	75		2	22	0.86	15		14	0.43	3					39	0.54			

*C. dewulfi*	46	1	3	27	0.44			14	0.43		1	1.00			4	0.25			1

*C. scoticus*	27		4	8	0.13	1		5	0.40		4	0.25			10	0.00			

*C. punctatus*	16		5	2	0.50						3	1.00		1	11	0.55			

*C. vexans*	15		6	9	0.89	3		2	1.00		3	1.00	2		1	1.00			

*C. achrayi*	10		7	2	0.50	1					1	0			7	0.57	1	2	

*C. chiopterus*	6		8	2	0.00			2	1.00		1	1.00			1	1.00			

*C. subfasciipennis*	5		9	5	0.80	1													

*C. pulicaris*	2	1	10												2	0.50			1

*C. picturatus*	1		11	1	1.00														

*C. circumscriptus*	1		11												1	1.00			

*C. santonicus*	1		11												1	1.00			

*C. shaklawensis*	1		11												1	1.00		1	

*C. simulator*	1		11												1	1.00		1	

*C. lupicaris*	1		11												1	0.00			

*C. clastrieri*		1	11																1

Obsoletus Complex	2		11					1	1.00						1	0.00			

*Culicoides sp*.	2			1	0.00						1	0.00							

**Total**	**525**	**9**		**232**		**70**	**4**	**74**		**4**	**83**		**2**	**8**	**136**		**1**	**4**	**5**

^1 ^For the sake of clarity, 0 were not quoted.

^2 ^F: females; M: males; E: engorged; G: gravide. Parity rate is No. parous/No. females.

In total, 232 females (44% of total catch) were collected with the drop trap, 136 (26%) by the UV-light/suction trap, 83 (16%) by the sticky cover trap and 74 (14%) by direct aspiration (Table 1). Among the host-baited traps, the sticky cover trap and the direct aspiration collected approximately the same number of species (7 with SCT and 6 with DA), whereas the drop trap collected 10 different species. The UV-light/suction trap collected 15 different species, of which *Culicoides circumscriptus *Kieffer, *Culicoides shaklawensis *Khalaf, *Culicoides simulator *Edwards, *Culicoides santonicus *Callot, Kremer, Rault & Bach or *Culicoides clastrieri *Callot, Kremer & Deduit were not collected with the other traps. The log abundance of the total number of females for each species was linearly correlated with the species rank (*R*^2 ^= 0.97; data not shown), with *Culicoides brunnicans *Edwards being the dominant species. The same shape of log abundance by species rank was observed for all the traps (*R*^2 ^= 0.86 for DT, 0.75 for DA and 0.76 for OVI), except for the sticky cover trap (*R*^2 ^= 0.51) due to the absence of *C. obsoletus *and the scarcity of *C. dewulfi *(Table 1). The Shannon-Wiener (*H*) and the Simpson-Yule (*D*) indices confirmed these differences: i) good correspondence between indices for the total number of females collected (*H *= 1.44 and *D *= 0.39), for drop trap (*H *= 1.22 and *D *= 0.46) and for direct aspiration (*H *= 1.42 and *D *= 0.31), ii) deviance with the sticky cover trap (*H *= 0.70 and *D *= 0.71) due to the under-representation of *C. obsoletus *and *C. dewulfi*, and iii) deviance with the UV-light/suction trap (*H *= 1.70 and *D *= 0.27) due to the under-representation of *C. dewulfi *and the over-representation of *Culicoides punctatus *(Meigen) and *Culicoides achrayi *Kettle & Lawson (Table 1).

Males were collected only by the drop trap and the UV-light/suction trap, and gravid females only by the sticky cover trap and the UV-light/suction trap (Table 1). Almost all engorged females were caught by the drop trap (70 *vs*. 4 by DA, 2 by SCT and 1 by OVI). We tested 44 blood-fed females (29 *C. brunnicans*, 14 *C. obsoletus *and 1 *C. scoticus*) of the 70 collected in the drop trap and 1 *C. brunnicans *of the 4 collected in the direct aspiration. All engorged females had fed on sheep except 2 *C. brunnicans *for which blood-meal origin was not identified. No blood engorged *C. dewulfi *was found, though it was the second dominant species in drop trap.

### Trap comparison

**Figure 3:**
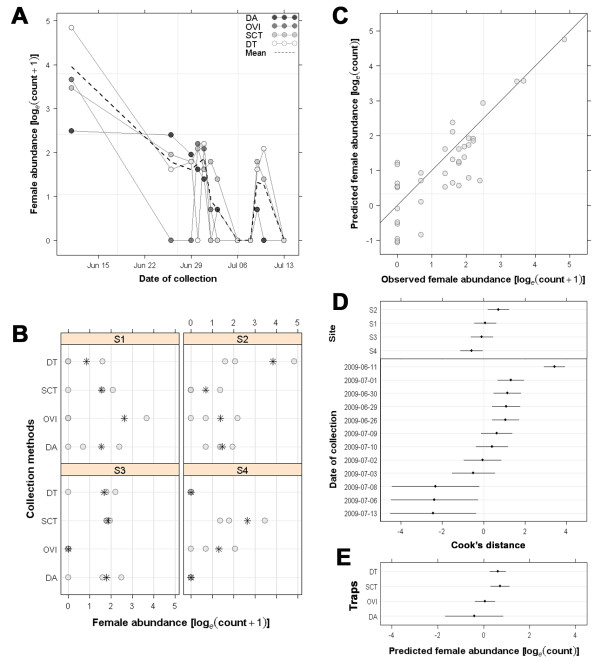
**Graphical exploration of *C. brunnicans *female collections**: (A) daily abundance, (B) number of females collected by site and by trap, (C) predicted and observed abundances, (D) assessment of the random effects of dates and sites and (E) predicted abundances by trap. NB: abundances were plotted using a log*_e _*scale.

The abundance of *C. brunnicans*, the dominant species, varied considerably between days (Figure 3A). Abundance of *C. brunnicans *and of other *Culicoides *species at the sites used during the studies varied (Figures 3A &3B), suggesting an impact of available larval habitat and suitable adult resting areas.

Abundance data for *C. brunnicans *were correctly predicted with the Poisson mixed model (*R^2 ^*= 0.98; Figure 3C). The same applied to the Hurdle model for *C. dewulfi*, *C. scoticus *and *C. punctatus*. Poor model predictions were obtained for *C. obsoletus*. Cook's distances showed influential values for site 2 where most *C. brunnicans *were collected and for site 4 where this species was nearly absent (Figure 3D). However, this variability was far more important for dates: the 11^th ^June recorded the highest abundance of *C. brunnicans *(Figure 3D), and the 6^th^, 8^th ^and 13^th ^the lowest abundances.

Both drop trap and sticky cover trap collected more *C. brunnicans *than the UV-light/suction trap whereas the direct aspiration was the less efficient method (*p *< 0.05) (Table 2). For other species, Wald test procedure gave unreliable results, *i.e*. p-values tend to 0 or 1, due to the low value of fitted probabilities [24,27]. Using the graphical procedure, differences were observed between traps for *C. obsoletus *abundance (*p *= 0.13): in positive collections, the UV-light/suction trap collected more *C. obsoletus *than drop trap or direct aspiration. It was not possible to establish differences between traps for *C. dewulfi *even if predicted abundances in positive collections were higher with the drop trap or the direct aspiration than with the UV-light/suction trap. Using graphical procedures, differences in predicted abundances were observed in positive collections for *C. scoticus *between sticky cover trap (the highest predicted abundance) and direct aspiration (the lowest) (*p *= 0.13). Finally, no between-trap difference was observed for *C. punctatus*.

**Table 2:** Observed and predicted biting rates per collection session

		Mean No. ♀				Predicted No. ♀^2^				
***Species***^1^	N	DT	DA	SCT	OVI	p	DT	DA	SCT	OVI
*C. brunnicans*	313	12.8	3.0	5.8	4.6	< 0.05	1.9^a^	0.7^b^	2.0^a^	1.1^c^

*C. obsoletus*	75	2.8	2.8	-	4.9	< 0.13	2.5^a^	2.6^a^	-	4.8^b^

*C. dewulfi*	46	3.9	2.0	1.0	1.3	-	3.8	1.6	-	0.6

*C. scoticus*	27	2.0	1.7	4.0	3.3	< 0.13	1.6^ab^	1.1^b^	3.9^a^	3.2^ab^

*C. punctatus*	16	2.0	-	3.0	1.8	-	1.6	-	2.8	1.4

^1 ^Data correspond to number of females per collection for *C. brunnicans *and to number of females per positive collection for the other species

^2 ^Different letters mean difference between traps in predicted number of females with the given p-value using the Wald test procedure for *C. brunnicans *and the graphical procedure for other species

The parity rate of *C. brunnicans *was higher in the UV-light/suction trap than in the host-baited traps (0.72 *versus *less than 0.65, p < 0.05) (Table 3). Parity rate of *C. obsoletus *was greater in the drop trap (0.86) than in UV-light/suction trap (0.54, p < 0.017) and in direct aspiration (0.86 in DT *versus *0.54 in OVI and 0.41 in SCT, p < 0.05). Finally, the low number of *C. punctatus *females did not allow us to compare parity rates between traps.

**Table 3:** Observed and predicted parity rate per collection session

		Observed parity rate				Predicted parity rate^1^			
*Species*	N	DT	DA	SCT	OVI	DT	DA	SCT	OVI
*C. brunnicans*	313	0.47	0.69	0.39	0.67	0.51^a^	0.65^a^	0.42^a^	0.72^b^

*C. obsoletus*	75	0.86	0.43	-	0.54	0.86^a^	0.41^b^	-	0.54^b^

*C. dewulfi*	46	0.44	0.43	1.00^2^	0.25	0.45	0.45	1.00	0.24

*C. scoticus*	27	0.12	0.40	0.25	0.00	0.12	0.40	0.25	0.00

*C. punctatus*	16	0.50	-	1.00	0.54	0.50	-	1.00	0.54

^1 ^Different letters mean difference between traps in predicted number of females for α = 0.05

^2 ^Only *C. dewulfi *parous female was collected with the sticky cover trap

### Preferential landing sites and circadian rhythm

**Figure 4:**
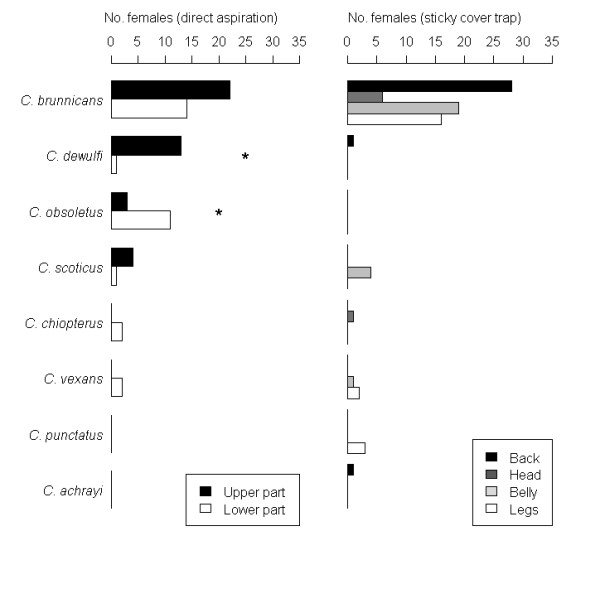
Number of females (for the 8 most abundant species) collected by direct aspiration and with the sticky cover trap on each animal body part (* *p *< 0.05).

By direct aspiration, we collected 57% of the females on the upper part of sheep and using the sticky cover trap 45% on the upper part (back and head). *Culicoides brunnicans *seemed to attack indifferently upper and lower parts of animal (Figure 4, *p *= 0.2 for DA and *p *= 0.9 for SCT). *Culicoides dewulfi *attacked the upper parts of the animal preferentially (*p *< 0.001 for DA), whereas *C. obsoletus *was more abundant on lower parts (*p *< 0.05 for DA). However, counts for both these species were relatively small limiting the generalization of these observations. Counts in other species were too small to highlight differences in attack zones (Figure 4).

**Figure 5:**
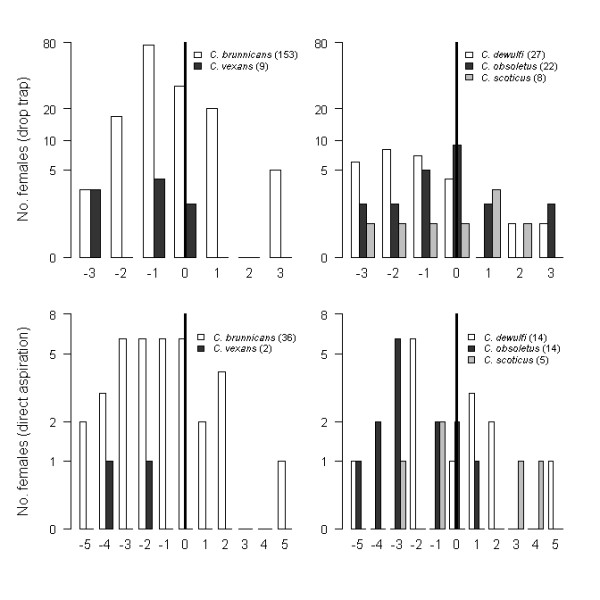
**Number of females (for the 5 most abundant species) collected by direct aspiration and with the drop trap each time period (20 min for DA and 30 min for DT) around the sunset (represented by thick line)**.

All Culicoides captured in the study exhibited a peak in activity cycle shortly before dusk (Figure 5).

## Discussion

This study has demonstrated for the first time that *C. brunnicans *can be present in substantial numbers on sheep and exceed the abundance of other commonly found *Culicoides *implicated in BTV within this region. In northern Europe, the most common species collected on livestock belong to the subgenus *Avaritia*: *C. obsoletus *and *C. dewulfi *on cattle [8,10], *C. obsoletus *on horses [9,11] and *C. obsoletus *and *C. chiopterus *on sheep [12]. All these species are suspected to be BTV8 vectors due to i) their abundance and their ability to bite ruminants, notably in absence of *C. imicola *[28], ii) the identification of BTV from field-collected individuals [29-31] and iii) for *C. obsoletus *and *C. scoticus*, their ability to be infected with BTV [5,32,33]. *Culicoides brunnicans *breeding sites are not clearly identified [34] but could include grasslands and, to a lesser extent, forests and wet areas (Delécolle, unpublished data). The high abundance of *C. brunnicans *may be related to the surrounding environment, which consisted primarily of pastures favorable to the species. *Culicoides brunnicans *is described as widely distributed in the western Palaeartic region [34]. Based on the French vector surveillance network, this species was collected in 73 out of 160 sites throughout the country. In these sites its abundance is usually very low: (1% of the total catch only in 23 sites) but locally abundant with an abundance reaching 10 to 13% of the total collection [35]. Little attention, however, has been paid to this species with respect to BTV transmission, despite its possible local abundance [36].

The risk of virus transmission is dependent, amongst other factors, on both biting rate and parity rate, a rough indicator of population survival [37-39]. Therefore, accurate and unbiased estimates of these parameters are essential for epidemiological modeling. However, collecting biting midges on animals is challenging, and *Culicoides *abundance may vary greatly between habitats [40]. In a given environment, flight activity will be greatly impacted by daily meteorological conditions, especially temperature, air humidity, light intensity or wind speed [12]. Moreover, host-seeking female distribution could be structured at a very fine scale [41], due to influence of host presence and abundance [42], or nearby environment [43,44]. Indeed, site 2 recorded the highest abundance of *C. brunnicans *probably because it was the nearest trap to the grazing sheep herd. For other, less abundant *Culicoides *species, the use of a single animal to bait traps may have limited the number of *Culicoides *caught [42]. Finally, observed parity rates were all rather high. It could mean that collections occurred at the end of the spring *Culicoides *activity peak and before the start of summer emergence of most species. This may lead to the low abundance recorded even if collections were carried out in temperature ranges compatible with *Culicoides *activity [45].

Direct aspiration was the only collection method which did not collect male or gravid females, suggesting that only host-seeking females were collected. This method collected fewer or equivalent numbers of females *versus *other host-baited traps, suggesting that human presence and activity during 10 min of aspiration may disturb host-seeking activity. Except for *C. scoticus*, the drop trap was the host-baited trap which collected the highest number of females. There were few males in the collections (due to the drop trap sampling air space near the host). This drop trap therefore does not strictly collect only host-seeking females, although they are most likely dominant among *Culicoides *collected in the vicinity of the host. There was also a higher proportion of parous *C. obsoletus *females compared to other traps. It is possible that the visual aspects of the drop trap (tall trap structure and the large white surface of the net) may have some effects. For instance, male swarm occurred more or less directly above an object contrasting strongly in color and brightness with the background [46]. This may lead to a possible bias in estimating the biting rate. One advantage of the drop trap is the possibility for *Culicoides *to feed quite naturally on animal bait. No *C. dewulfi *females were found engorged on sheep, suggesting that this species might be attracted by sheep without feeding on them. The same behavior was suggested recently for *C. chiopterus *[19], both species being recognized to be strongly associated with cattle [47]. The sticky cover trap collected the same abundance and parity rate of *C. brunnicans *than the drop trap, thus suggesting that this trap was efficient to estimate biting rate for dominant species, even if presence of gravid or engorged females may suggest that it could act as an interception trap. Surprisingly, the sticky cover trap did not collect any *C. obsoletus *and only one *C. dewulfi*. These species could attack animal parts which were not sufficiently covered by the sticky cover (i.e. head, ears). However, we cannot exclude a repellent effect of the petroleum jelly to these species, even if it is probable that *Culicoides *could not detect the gel before entering into contact because this product does not evaporate. This trap may be improved by a better design and by using another sticky substance easier to handle than petroleum jelly.

In our study, the UV-light/suction trap under-estimated the *C. brunnicans *biting rates, whereas it seemed to over-estimate *C. obsoletus *biting rates as recorded by Carpenter *et al*. [12]. It can be in contradiction to Gerry *et al*. [13] who collected fewer *C. obsoletus *females in the UV-light/suction trap than on sheep but these apparently contradictory findings might be explained by the very high suction rate (air flow) of the OVI relative to the CDC-type traps used by Gerry *et al*. [13,14]. This trap remains an efficient and practical tool to describe species richness in an area, but presence of males, gravid females and single specimens of species which were not collected by host-baited traps suggested that this method did not only collect host-seeking females in the UV-light/suction trap. This could undermine the assessment of BTV risk as UV-light/suction traps have been used in national surveillance networks since 2000 in south Europe and since 2008 in north and central of Europe, most *Culicoides *survey were done using UV-light/suction traps [28,48-51].

Collections by direct aspiration and sticky cover trap highlighted that *C. brunnicans *attacked all parts of the animal, whereas *C. obsoletus *seemed to attack preferentially lower parts and *C. dewulfi *the upper parts. Nielsen [8] found the same behavior for *C. obsoletus*, with nearly all individuals collected from the belly of heifers, whereas *C. chiopterus *attacked preferentially the legs. On the contrary, Townley [9], who explored the preferential landing and feeding sites of *Culicoides *on horses in Ireland, observed that the most abundant species, *C. obsoletus *and *C. dewulfi*, did not seem to have preferential landing sites whereas *C. punctatus*, *Culicoides nubeculosus *(Meigen) and *Culicoides pulicaris *(Linnaeus) fed at the predilection sites of sweet itch. Preferential landing sites may differ for a same *Culicoides *species depending on the host, due to variations in hair wool density, colors and skin temperatures according to the host body parts [52].

For the first time in Europe, this study compares the ability of multiple types of host-baited traps to collect host-seeking females compared to UV-light/suction traps. We observed bias of each trapping method through *Culicoides *sampling, which highlight the relevance of each trap for different kinds of *Culicoides *ecology studies. Direct aspiration seemed to collect only host-seeking females and can be used to assess accurately *Culicoides *biting rate even if the possible disturbance of host-seeking females due to human manipulation is not clear. The sticky cover trap showed its ability to assess biting rates of abundant species. After improvements (better design of the cover or sticky substance easier to use), this method has promise to conduct easily (without human intervention) host-baited collections even if we do not have an explanation of the *C. obsoletus *absence in collections. The drop trap resulted in higher estimated *Culicoides *biting rates or numbers (including presence of males in collections, high abundance for most host-biting species). Most importantly, the main advantage of the DT is that it allows assessing the engorgement level of insects and then highlighted singular behavior as for *C. dewulfi*, which seems attracted to sheep but unwilling to feed on them. The UV-light/suction trap is the most effective method to collect large numbers of *Culicoides *midges, for example to carry out biological studies involving live *Culicoides *midges in the absence of a colony, because it remains the easiest to use maximizing diversity in collections. However, UV-light/suction traps abundances cannot be used directly to assess *Culicoides *biting rates. Given possible environmental influences on *Culicoides *species behaviors, this study should be repeated in other ecosystems hosting other species and/or other hosts (horses, cattle) and in different climatic conditions to obtain a better understanding of the relation between biting rates and UV-light/suction trap collections. This is of significant importance for the assessment of BTV risk throughout Europe.

## Competing interests

The authors declare that they have no competing interests.

## Authors' contributions

EV, CG and TB designed the study. EV, CG, TB, XA, LG, IR and DC carried out the experimentations. EV, RL and TB analyzed the data. EV and TB wrote the manuscript, which was revised by CG, TB, CM, and RL. All authors read and approved the final manuscript.
